# Report of a Case of Membranous Croup Successfully Treated with O’Dwyer’s Tubes

**Published:** 1898-05

**Authors:** Jno. W. Kenney

**Affiliations:** San Antonio, Texas


For the Texas Medical Journal

### Report of a Case of Membranous Croup Successfully Treated With O'Dwyer's Tubes.

BY JNO. W. KENNEY, M. D., SAN ANTONIO, TEXAS.

In reporting this case of membranous croup it is not my intention to enter into a discussion of the ætiology or pathology of the affection. Neither is it my purpose to take issue with those who consider diphtheria and membranous croup as one and the same disease or with those who deem them separate and distinct. Will leave that to those whose time, attention and deliberations have been given to laboratory investigation. At best my opinion could be but empirically given, therefore I refrain from expressing it.

I shall endeavor to report the case as it occurred in my practice, together with the simple treatment and the happy results obtained thereby.

The patient was a girl, aged two and one-half years, fairly well nourished, and had been ailing four days. The mother gave as the probable cause of the trouble the fact that she had given the child a cold shower bath on a damp and chilly day. If there was a source of infection, it could not be ascertained. A history of increasing hoarseness as evening approached, caused me to direct my attention to the throat. Inspection, however, revealed nothing abnormal. There was no elevation of temperature, but slight acceleration of pulse, and the only evidence of constitutional disturbance perceptible was the hoarseness and slight restlessness.

The insidiousness of the trouble caused me to look upon it with suspicion, and therefore devote to it considerable attention.

An emetic was prescribed, to be used during the night, should an emergency arise. Quinine was given in small doses, as a placebo, and the future course of the trouble awaited. During the next two days there was not any material change in the condition of the patient, unless it be that the hoarseness was a little more pronounced. The following day (Sunday) there was developed marked difficulty in breathing, which by the middle of the afternoon became so pronounced that death seemed pending. Dr. Eugene Clark was called in consultation, and it was decided that intubation with O'Dwyer's tubes be practiced, in preference to tracheotomy. The child's face was livid, and its gasping and, I might say, grasping for air, for it was tossing itself about and pulling at everything its hands by chance touched, was pitiable in the extreme.

The mouth gag was placed in position, and in a few seconds the tube was inserted by Dr. Clark into the larynx after a little strangling and coughing, during which a quantity of mucous and shreds of necrotic tissue were expelled, the breathing became easy, the dusky hue departed from the patient's face—the restlessness passed away, and in half an hour the child was asleep and breathing as easily as anyone in perfect health.

A silk thread about two feet in length was attached to the tube before insertion. This was left hanging from the mouth to guard against the swallowing of the tube should it by accident be coughed up, and also to facilitate its removal.

Monday morning I was called in hastily, and to my chagrin found that the tube had been coughed up and swallowed, string and all. The breathing, however, was easier, but only for a short time. Two hours later I placed a larger sized tube in the larynx with the same happy result as before. This time the precaution was taken to sew the thread to the child's clothing, thereby precluding the possibility of a recurrence of the accident that had happened with the former tube. The tubes are a little too expensive to feed to children, and then, too, there is a possibility of some injury resulting to the child from the effects of its swallowing such an indigestible bolus.

In this case, however, the tube, with thread attached, passed by the lower bowel the following evening but little the worse for its alimentary journey and without any noticeable injury to the little patient.

On the following Saturday I found my patient sitting up in bed playing with its toys and seemingly at ease. There was a slight elevation in temperature—about one degree. The tube was removed but had to be again replaced in about eight hours. This time considerable difficulty was experienced in introducing the tube. This time I allowed it to remain seven days when it was again removed—no further trouble was experienced and the convalescence was rapid. It was some time, however, after the removal of the tube before the voice became normal. Nature looked well to this without the aid of medicament.

During the last few days of the child's illness its temperature ranged from 101° F. to 102° F. This I attribute to the absorption of poisonous products in the ulcerated larynx—an acute pyamia, as it were. Two grains of mild chloride of mercury were prescribed in quarter grain doses, which acted well and was of perceptible benefit.

After becoming accustomed to the tube the little patient rested well and experienced but little inconvenience. The mother, however, was annoyed and uneasy, and was exceedingly anxious that medicine should be given her child. Accordingly I prescribed a proprietary drug that is claimed to promote the formation of phagocytes and thereby answer as a specific for all microbic diseases. Whether it was of any service to my patient or not I am unable to state, but of one thing I am sure, and that is that it worked like a charm upon the mother and allowed her to live easy. I justify myself in doing this for the same reason that one is justified in giving anodynes to produce euthanasia. It is certainly more important that we should live easy than that we should die without pain, for as it is somewhere said: "The living we have with us always."

In a comparison of the relative merits of tracheotomy and intubation, I think that intubation possesses a decided advantage. Aside from the few cases in which intubation cannot be practiced, I see but little ground for the making of an artificial opening into the trachea or larynx. Less skill is required in the performance of tracheotomy, and it can be done in emergencies when the tubes are not at hand, and without assistants. The objections to it are many. In the first place, any cutting operation is revolting to the parents; the danger of pneumonitis is great from the inhalation of the raw or cold air; the dressing of the wound and the time required for healing, together with the resulting eschar, are anything but desirable. The mortality after this operation is almost one hundred per cent., a fact that presents but little encouragement.

The greatest objection that can be offered to the intubation method of treating laryngeal stenosis resulting from membranous croup, is that considerable difficulty is sometimes experienced by unknowing hands in feeding the patient. Here nature comes in for some little censure for what appears to be a bad anatomical arrangement, for, were the œsophagus placed anterior to the trachea, this trouble would be obviated. That some skill is required to place the tubes in the larynx, cannot be offered as an objection, for we are all supposed to be skilled men in the profession which we represent. The advantages of intubation are manifold—the use of the knife is dispensed with, and with it the healing process and eschar; the air is warmed and moistened before entering the lungs; the shock is decidedly less than after tracheotomy; the use of an anæsthetic is not necessary; and finally, the consent of parents is easily gained for this operation, and the mortality is, according to the best authorities, at least twenty-five per cent. less than after tracheotomy.

After these tubes have been fixed in the larynx, and all the while that they remain in situ, the same skilled and watchful nursing is required as after tracheotomy. If at any time the hymen of the tube should become obstructed the nurse should remove the instrument by making traction on the string. In view of the possibility of this accident I deem it advisable to leave the string attached to the tube. But few nurses could use the extractor in such an emergency, and the tickling sensation at first produced by the string soon passes away. In feeding the child the head should be placed lower than the body and tilted to one side. Solid food is preferred by some physicians, but in my case liquids served better. When the child will not use a nursing bottle it may be fed with a spoon. The liquid should be poured slowly into the mouth and allowed to flow along the buccal mucous membrane to the œsophagus.

The after treatment of this disease is equally as important as the treatment of the trouble in its reality. The child's entire economy has become debilitated, and likewise the heart is weakened. It is with difficulty that it performs its normal function, and should any undue labor be placed upon it the danger of its failing is great.

Often just as we are elated that our patient is out of danger the news reaches us of its sudden death. This should not be such a common occurrence, for, as it is well known to us all, there should be provision made to fight against such an untoward finale.

In the case that it has just been my privilege to report my instructions were that a little whiskey be given five or six times during the twenty-four hours for ten or twelve days. This served its purpose well in this one instance, at least.

In advocating intubation in preference to tracheotomy, I do so not simply with the zeal of a recent convert, but for the common sense advantages it possesses as I see them. That it will ever entirely supercede tracheotomy as a treatment for laryngeal stenosis I do not maintain, for there are cases in which one or even both may have to be resorted to that life may be saved.

In children over five years of age the O'Dwyer tubes do not seem to act so well, presumably on account of the strength and control which the patient has over the hyoglossus and stylopharyngeus and other muscles of the post lingual and pharyngeal regions. Possibly the ingenuity of some one may enable him to furnish us with a tube that will more perfectly adapt itself to the larynx of a child at this age.

Since the successful use of the tubes in the above case, my friend, Dr. A. S. McDaniel, in his extensive practice, has had occasion to use them in several cases. His experience tallies with my own, for in every instance in which they were used in young children—that is, children under five years of age—the patient was tided over the crisis, and a most happy result obtained. In the doctor's cases, however, he could not resist the temptation to administer the antitoxin. Parenthetically, I will state that the antitoxin was used on some of those who died when the tubes could not be used—the case in mind being that of a boy nine years of age, who could not retain the tube more than a few hours at a time—just long enough to recover from the semi-asphyxiated state into which he invariably relapsed after expelling the tube.

